# Social Contact Prior to COVID-19 and Longitudinal Mental Health Trajectories During COVID-19 Among Adults Ages ≥55

**DOI:** 10.1093/geroni/igab046.1231

**Published:** 2021-12-17

**Authors:** Daniel R Y Gan, John Best, Jessica Finlay

**Affiliations:** 1 Simon Fraser University, Vancouver, British Columbia, Canada; 2 University of Michigan, Ann Arbor, Michigan, United States

## Abstract

Social support protects mental health during a crisis. We examined whether prior contact with social organizations and friends/neighbors was associated with better trajectories of loneliness, depression and self-rated memory during the COVID-19 pandemic. We conducted latent class analysis and regression analysis on longitudinal data from the COVID-19 Coping Study of US adults aged ≥55 from April-October 2020 (n=3105). Overall, prior contact with friends(B=-.075,p<.001), neighbors(B=-.048,p=.007), and social organizations(B=-.073,p<.001) predicted better mental health amid COVID-19. Three classes were identified: Class1 had the best outcomes, whereas Class3 had the worst outcomes and were most likely to live alone(B=.149,p<.001). For Class1, prior contact with social organizations(B=-.052,p=.044) predicted decreasing loneliness. For Class2, prior contact with friends(B=-.075,p<.001) predicted decreasing loneliness and better memory(B=-.130,p=.011). Conversely, prior contact with neighbors(B=-.165,p=.010) predicted worsening loneliness among Class3. Our findings pose new questions on the role of neighborhood networks to mitigate poor mental health outcomes among older adults during a crisis.

